# Longitudinal follow-up of the asthma status in a French–Canadian cohort

**DOI:** 10.1038/s41598-022-17959-6

**Published:** 2022-08-13

**Authors:** Marie-Eve Lavoie, Jolyane Meloche, Anne-Marie Boucher-Lafleur, Paul Bégin, Charles Morin, Louis-Philippe Boulet, Anne-Marie Madore, Catherine Laprise

**Affiliations:** 1 Centre Intersectoriel en Santé Durable, Université du Québec à Chicoutimi, Saguenay, QC G7H 2B1 Canada; 2 Département des Sciences Fondamentales, Université du Québec à Chicoutimi, Chicoutimi, Saguenay, QC G7H 2B1 Canada; 3 Centre Intégré Universitaire en Santé et Services Sociaux du Saguenay–Lac-Saint-Jean, Saguenay, QC G7H 7K9 Canada; 4 Centre de Recherche de l’Institut Universitaire de Cardiologie et de Pneumologie de Québec (CRIUCPQ), Université Laval, Québec, QC G1V 4G5 Canada

**Keywords:** Medical research, Risk factors

## Abstract

Asthma affects 340 million people worldwide and varies in time. Twenty years ago, in Canada, the Saguenay–Lac-Saint-Jean asthma family cohort was created to study the genetic and environmental components of asthma. This study is a follow-up of 125 participants of this cohort to explore the appearance, persistence, and progression of asthma over 10–20 years. Participants answered a clinical standardized questionnaire. Lung function was assessed (forced expiratory volume in 1 s, forced vital capacity, bronchial reversibility, and methacholine bronchoprovocation), skin allergy testing was performed, blood samples were obtained (immunoglobulin E, white blood cell counts) and phenotypes were compared between recruitment and follow-up. From the participants without asthma at recruitment, 12% developed a phenotype of adult-onset asthma with the presence of risk factors, such as atopy, high body mass index, and exposure to smoking. A decrease of PC_20_ values in this group was observed and a decrease in the FEV_1_/FVC ratio in all groups. Also, 7% of individuals with asthma at recruitment developed chronic obstructive pulmonary disease, presenting risk factors at recruitment, such as moderate-to-severe bronchial hyperresponsiveness, exposure to smoking, and asthma. This study allowed a better interpretation of the evolution of asthma. Fine phenotypic characterization is the first step for meaningful genetic and epigenetic studies.

## Introduction

Nearly 340 million people around the world were affected by asthma according to the Global Burden of Disease Study in 2016^1^, making it one of the most common chronic diseases worldwide. In Canada, 3.8 million people live with asthma^2^. The prevalence of asthma continues to increase in low-to-middle-income countries, whereas in high-income countries it seems to have peaked because of improved asthma control or reduced incidence^3^.

Asthma is a chronic inflammatory disease of the respiratory tract characterized by airway hyperresponsiveness (AHR) and airway obstruction^4^. It can affect people of all ages and vary over time. Some people develop asthma during childhood and see their symptoms disappear in their teenage years, while others develop the disease in adulthood^5^. These symptoms can be triggered by several factors, such as exposure to allergens in sensitized people and irritating agents (smoke, strong smells, or exhaust fumes), exercising, and changing seasons, as well as respiratory infections^4^. Environmental stimuli may trigger an inflammatory cascade driven by T-helper type 2 (T_H_2) cells in asthmatic patients^6,7^, leading to narrowing of bronchi lumen, which is reflected by a decrease in forced expiratory volume in 1 s (FEV_1_) in spirometry testing. AHR can be estimated by the PC_20_, the methacholine concentration inducing a 20% decrease in FEV_1_. Furthermore, persistent airflow limitation, defined as a reduced ratio of FEV_1_ to forced vital capacity (FVC) post-bronchodilator, can develop in a subgroup of patients with adult-onset asthma^8^. A reduced FEV_1_ to FVC ratio (below 0.70) is also observed in chronic obstructive pulmonary disease (COPD)^9^.

Asthma is a multifactorial disorder. Since asthma development and progression are influenced by various genetic and environmental factors, the pathogenesis and natural history of this disease are not well understood^10^. Longitudinal studies are required to discover the evolution of asthma over time^11^. Many studies have investigated the atopic march and featured childhood asthma, which helped establish a better picture of the situation^12,13^. An improved characterization of the various phenotypes in adults would facilitate a greater understanding of the clinical evolution of the disease and its persistence, create a better representation of asthma in an adult population over time, and may identify new therapeutic strategies.

**Figure 1:**
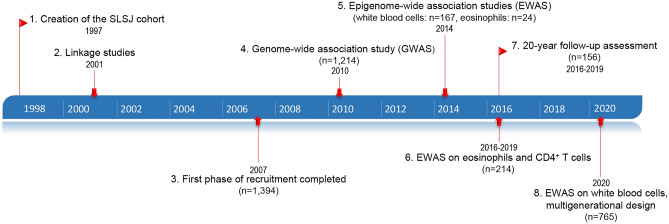
Timeline of the SLSJ asthma family cohort. Stages in the acquisition of phenotypic, genomic, and epigenomic data for the SLSJ family cohort. Description of approaches used at each stage: 1.–3. Standardized questionnaire, respiratory measurements, blood samples (IgE, differential white blood cell counts, and DNA), 2. Microsatellite markers, 4. Illumina 610 K-Quad chip, 5. Infinium Human Methylation 450 K chip (white blood cells and eosinophils), 6. Isolation of naive CD4^+^ T cells and eosinophils for DNA and RNA extraction for methylome and transcriptome (methylome sequencing with custom methyl capture panel [MCC-Seq] and RNA sequencing), 7. Standardized questionnaire, respiratory measurements, blood samples (IgE, differential white blood cell counts, DNA, RNA, and plasma), 8. MCC-Seq (white blood cells). Between 2001 and 2010, candidate gene studies were performed with genetic variants, expression, and methylation data.

Recruitment to the Saguenay–Lac-Saint-Jean (SLSJ) asthma family cohort began 20 years ago to study the influence of the genetic determinants on asthma and its persistence over time^14^ (Fig. 1). Since we focused on the genetics of allergic asthma, we recruited affected probands and their parents, with the consequence that the majority of the asthmatic individuals are allergic and have mild-to-moderate asthma. The present study evaluates the respiratory condition of a subsample of the SLSJ asthma family cohort, using the same clinical procedures (health questionnaire, spirometry, and skin prick test) to study the evolution of the disease 10–20 years after their first visit. The objective of this study was to assess changes in asthma status and other respiratory or clinical parameters.

## Methods

### Study population

The first phase of recruitment for the SLSJ cohort was from 1997 to 2003 and 2006–2007. It consisted of 1394 individuals from 271 independent families^14^. Recruitment was conducted through probands and then included all family members who wanted to participate in the study. Additional detail on recruitment is provided in the Supplementary File 1 (in the study population section). The present study was realized 20 years after the first recruitment phase (from 2016 to 2019). A total of 215 participants were selected in the context of another project and the follow-ups were done at the same time. There were initially 156 participants in the follow-up research project. Among them, 31 participants were children at recruitment, i.e., 16 years old and younger (the age corresponding to adult asthma). The remaining 125 individuals (80%), comprising 58 men and 67 women, agreed to participate in the two required visits to reassess their respiratory condition. Thus, only the adult participants at recruitment and follow-up were kept to perform analyses on an adult population only, therefore preventing mixing of children and adult clinical measures. Informed consent was obtain from all participants and/or their legal guardians at recruitment, in accordance with the rules of the ethics committee of the *Centre intégré universitaire de santé et de services sociaux (CIUSSS) du Saguenay‒Lac-Saint-Jean* (project #0002-001). This committee also approved experimental protocols including questionnaires, respiratory measures and skin prick tests.

### Measurements and definitions of outcomes

In this longitudinal study, a clinical standardized respiratory questionnaire was completed (modified from^15^). The questionnaire evaluated many elements regarding asthma and related phenotypes that are self-reported such as age of onset of asthma, allergies, respiratory symptoms, medication, health habits, family history, environmental exposures, and comorbidities. Thereafter, respiratory measurements were carried out according to the guidelines of the American Thoracic Society^16^. FEV_1_, PC_20_ and bronchodilator (BD) reversibility test were performed to assess lung function parameters^14^. Allergy was assessed using skin prick test for 27 allergens including animal dander, indoor and outdoor aeroallergens, as well as food allergens (see “Supplementary File” in the data collection section). Participants were considered atopic if they exhibited at least one positive response as defined by a wheal diameter ≥ 3 mm and > 50% of positive control (histamine) reaction after 10 min. Blood samples were collected from each participant to measure serum IgE levels, white blood cell counts (basophils, eosinophils, lymphocytes, monocytes, and neutrophils), as well as to perform DNA and RNA extraction and collect plasma samples for further studies^14^. Data were also collected at the archives of Chicoutimi hospital (*CIUSSS*) for 123 of the 125 participants; two of them did not have records at this hospital. Information on asthma and COPD diagnosis were considered. Only data from the standardized respiratory questionnaire and clinical assessment at follow-up were used for the two participants with no records at the *CIUSSS.*

### Statistical analyses

Statistical analyses were performed using the SPSS Statistics 25.0 (IBM SPSS Statistics, Version 25.0. Armonk, NY: IBM Corp). Normal distribution was verified for all groups tested, and parametric or non-parametric tests were performed accordingly. A log_10_- transformation was used to render total serum IgE levels and PC_20_ concentration distributions approximately normal. To analyze the differences between groups at recruitment and follow-up, T-test and Wilcoxon test for matched data were conducted for continuous variables and Chi-square or Fisher’s exact test for categorical data. In Table 1, the differences were measured for each group, i.e., the All participants, the No asthma, and the Asthma groups, between recruitment and follow-up and thus for each parameter. Analyses were performed in order to measure possible associations between variables. Spearman Rho correlations were used between all variables. Multiple linear regression models were also tested to identify variables explaining significant differences between recruitment and follow-up that were found in previous analyses, with models having identified variable with significant differences as dependent variable and values of likely explanatory variables at first recruitment as independent variables. P-values < 0.05 were considered significant.

**Table 1:** Characteristics of the 125 individuals at recruitment and follow-up.

Parameters	Recruitment			Follow-up		
	All participants (n = 125)	No asthma (n = 57)	Asthma (n = 68)	All participants* (n = 125)	No asthma* (n = 57)	Asthma* (n = 68)
Age, mean (range)	41 (17–68)^†††^	43 (17–68)^†††^	39 (17–62)^†††^	58 (27–83) ^†††^	60 (27–83)^†††^	56 (29–81)^†††^
Sex M:F (ratio)	1:1.16	1:1.04	1:1.27	1:1.16	1:1.04	1:1.27
Asthma, n (%)**	68 (54)	0 (0)^†^	68 (100)	75 (60)	7 (12)^†^	68 (100)
Age of onset, mean (range)	17 (0–54)	NA	17 (0–54)	19 (0–64)	43 (17–64)	17 (0–54)
FEV_1_, %pred pre-BD (SD)	95 (18)	102 (15)	89 (17)	94 (20)	103 (16)	87 (21)
FEV_1_/FVC pre-BD (SD)	0.78 (0.09)^†††^	0.81 (0.06)^†††^	0.75 (0.10)^††^	0.74 (0.09)^†††^	0.78 (0.06)^†††^	0.71 (0.10)^††^
FEV_1_/FVC post-BD (SD)	0.80 (0.08)^†††^	0.83 (0.05)^†††^	0.77 (0.10)^††^	0.77 (0.08)^†††^	0.80 (0.05)^†††^	0.73 (0.09)^††^
PC_20_, mg/ml (SD)	10.44 (5.9)	28.07 (2.9)^†^	3.53 (5.8)	9.93 (5.8)	19.75 (3.9)^†^	4.68 (6.3)
BD, % (SD)	6.21 (6.5)	3.79 (4.8)	8.06 (7.1)	5.51 (8.4)	3.25 (4.4)	7.24 (10.1)
IgE, μg/L (SD)	103.08 (4.4)	56.31 (3.7)	168.50 (4.2)	106.37 (4.1)	66.24 (3.5)	156.24 (4.1)
**White blood cell count, % (SD)**						
Eosinophils	3.16 (2.14)^†^	2.47 (1.35)	3.74 (2.50)	3.67 (3.15)^†^	2.81 (2.32)	4.39 (3.56)
Lymphocytes	29.06 (7.07)	31.05 (6.11)^†^	27.41 (7.43)	27.95 (7.49)	28.81 (6.80)^†^	27.24 (7.99)
Monocytes	7.82 (2.18)	7.56 (1.66)	8.04 (2.52)	8.49 (4.53)	8.23 (6.00)	8.71 (2.83)
Neutrophils	59.34 (8.12)	58.29 (6.74)	60.21 (9.08)	59.73 (8.45)	59.88 (6.66)	59.60 (9.74)
Basophils	0.77 (0.58)	0.61 (0.52)	0.91 (0.60)^†^	0.66 (0.48)	0.65 (0.48)	0.67 (0.47)^†^
BMI, (SD)	24.96 (3.71)^†††^	25.01 (3.13)^††^	24.92 (4.12)^†††^	27.06 (5.81)^†††^	27.10 (4.72)^††^	27.03 (6.57)^†††^
Smoking status, n (%)**						
Non-smoker	60 (49)	24 (43)	36 (55)	45 (42)	17 (35)	28 (48)
Ex-smoker	41 (33)^††^	20 (36)^†^	21 (31)^†^	58 (54)^††^	28 (58)^†^	30 (51)^†^
Smoker	22 (18)^††^	12 (21)^†^	10 (15)^†^	4 (4)^††^	3 (6)^†^	1 (2)^†^
Atopy, n (%)**	75 (60)	25 (44)	50 (74)	54 (52)	23 (46)	31 (57)
Rhinitis, n (%)**	50 (40)^†^	18 (32)	32 (47)^†^	27 (24)^†^	9 (17)	18 (30)^†^
Atopic dermatitis, n (%)**	58 (46)	28 (49)	30 (44)	51 (44)	21 (40)	30 (48)
COPD, n (%)	0	0	0	5 (4)	0	5 (7)

Data is shown as mean (SD), n (%) or in range.

Statistics were performed for each group, i.e., the All participants, the No asthma, and the Asthma groups, between recruitment and follow-up, using T-test or Wilcoxon test for matched data or continuous variables or chi-square or Fisher exact test for categorical variables: †p** < **0.05, ††p < 0.001 and †††p < 0.0001.

*BMI* body mass index (kg/m^2^), *BD* bronchodilator response, *COPD* chronic obstructive pulmonary disease, *FEV*_*1*_ forced expiratory volume in 1 s, *FVC* forced vital capacity, *IgE* immunoglobulin E, *NA* not available, *PC*_*20*_ provocative concentration for a 20% decrease in FEV_1_, *SD* standard deviation.

*Same individuals as at recruitment.

**Percentage calculated with available data only (does not include missing values).

### Ethics declarations

All participants provided written consent for their participation, in accordance with the rules of the ethics committee of the *Centre intégré universitaire de santé et de services sociaux (CIUSSS) du Saguenay‒Lac-Saint-Jean* (project #0002-001).

## Results

Table 1 presents the phenotypic description of the participants at recruitment and at follow-up and is divided into three groups (All participants, No asthma, and Asthma) according to the asthma status of participants at recruitment. The subsample of 125 participants included 58 men and 67 women. At recruitment, the age of these individuals ranged from 17 to 68 years (mean of 41 years). At follow-up, participants were aged 27–83 years (mean, 58 years). BMI values increased from 24.96 at recruitment to 27.06 at follow-up (p < 0.0001) and the number of smokers displayed a marked decrease at follow-up from 22 to four participants (p < 0.001).

### Changes of asthma phenotype

As depicted in Table 1, 54% of the individuals, i.e., 30 men and 38 women, had asthma at recruitment. Based on asthma diagnoses validated by a physician, three women and four men developed asthma between recruitment and follow-up, all of them developing adult-onset asthma (> 16 years-old). This is a 12% status change among the 57 participants without asthma at recruitment. The mean age of onset was 43 years (see the No asthma group of the follow-up section in Table 1). They included two men and two women with obesity (BMI > 30), five smokers or ex-smokers and five participants with atopy (Table 2). In addition, three individuals observed the disappearance of asthma symptoms during the same period; however, they are still considered to have asthma as the individuals who revert to a non-asthmatic phenotype and never experience asthma symptoms again are rare (Table 3)^5^.

**Table 2:** Changes in the respiratory status of seven participants who developed asthma between recruitment and follow-up evaluation.

	Sex	Age	BMI	Atopy	Smoker	PC_20_	BD %	FEV_1_, %pred	FEV_1_/FVC
		Recruitment/follow-up							
1	F	17/27	25.10/40.09	Yes/yes	N-S/N-S	4.09/4.67	0/3.33	109/90	0.83/0.85
2	F	40/50	33.72/35.00	Yes/yes	E-S/E-S	1.22/0.125	5.26/10.80	95/83	0.85/0.82
3	M	52/71	25.76/25.76	Yes/yes	E-S/E-S	12.90/64.00	NA/7.30	86/96	0.76/0.78
4	M	51/70	29.33/38.89	No/no	S/E-S	65.00/30.52	0.89/0	112/106	0.88/0.85
5	M	44/64	23.04/25.20	No/no	S/E-S	66.00/NA	5.43/9.48	92/87	0.78/0.75
6	M	29/48	26.70/30.14	Yes/yes	N-S/N-S	10.80/1.39	8.08/18.75	99/80	0.83/0.75
7	F	18/36	25.56/26.17	No/yes	S/S	4.00/2.78	9.17/15.56	109/90	0.89/0.80

*BMI* body mass index (kg/m^2^), *BD* bronchodilator response, *E-S* ex-smoker, *FEV*_*1*_ forced expiratory volume in 1 s, *FVC* forced vital capacity, *N-S* non-smoker, *NA* not available, *PC*_*20*_ provocative concentration for a 20% decrease in FEV_1_, *S* smoker.

**Table 3:** Parameters of the three participants who reported a disappearance of asthma symptoms at follow-up.

	Sex	Asthma	Age	BMI	Atopy	Smoker	PC_20_	BD %	FEV_1_, % pred	FEV_1_/FVC
		Recruitment	Recruitment/follow-up							
1	M	Yes	43/58	23.67/24.88	Yes/yes	E-S/E-S	8.87/1.05	0/17.14	107/105	0.68/0.66
2	F	Yes	46/64	24.34/23.53	Yes/yes	N-S/E-S	49.10/52.71	0/4.29	90/70	0.77/0.72
3	M	Yes	45/65	28.41/32.22	No/no	E-S/E-S	11.5/NA	3.17/3.81	98/105	0.83/0.73

*BMI* body mass index (kg/m^2^), *BD* bronchodilator response, *E-S* ex-smoker, *FEV*_*1*_ forced expiratory volume in 1 s, *FVC* forced vital capacity, *N-S* non-smoker, *NA* not available, *PC*_*20*_ provocative concentration for a 20% decrease in FEV_1_, *S* smoker.

**Figure 2:**
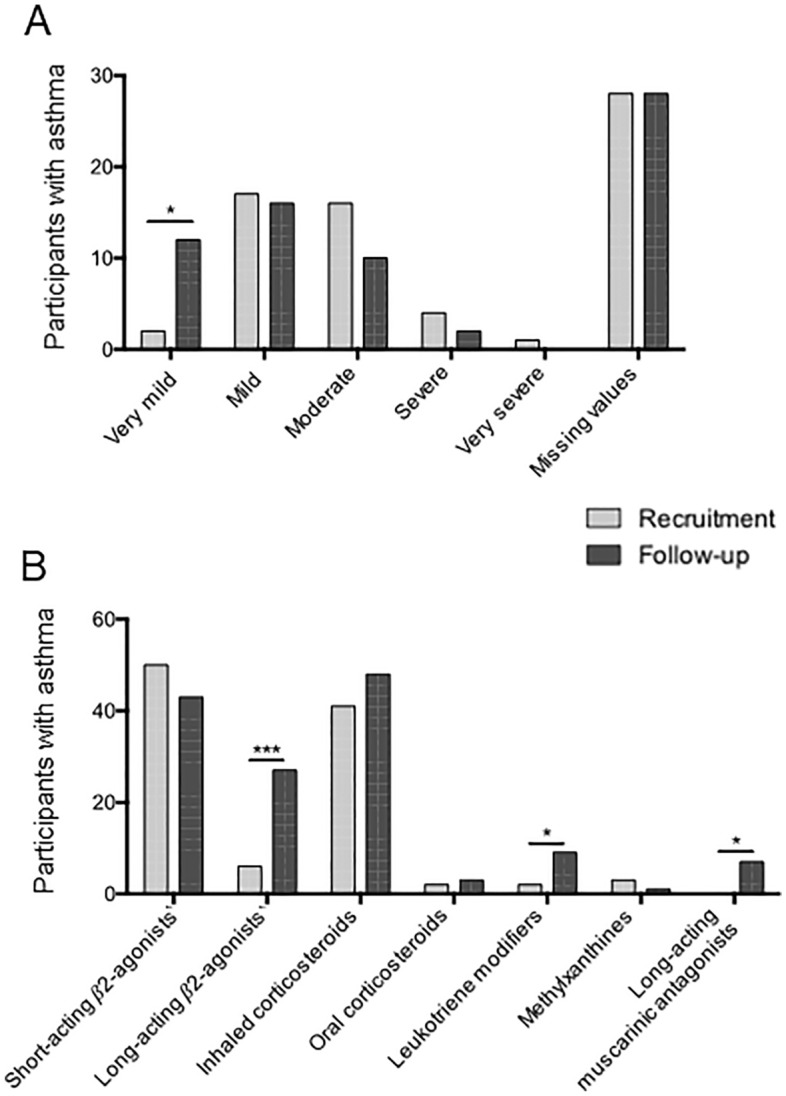
Asthma severity over time. (**A**) Participants classified the severity of their disease using a self-reported questionnaire. (**B**) The use of prescription medication for asthma control evolved between recruitment and follow-up. p values < 0.05 are indicated by an asterisk and p values < 0.0001 are indicated by three asterisks.

Changes have been observed in different indices of asthma severity. The severity of asthma from very mild to very severe was compared based on data collected using the questionnaire during the follow-up period. Between recruitment and follow-up, there was an increase in the number of participants who characterized their asthma as being very mild, from two to 12 individuals (χ^2^ p < 0.05, Fig. 2A and Supplementary Table S1). Amongst the individuals with new very mild asthma status, six categorized their asthma as mild, four as moderate, and the others had missing values at recruitment. In contrast, a decrease in the number of participants in the moderate asthma category was observed, from 16 to 10 individuals (χ^2^ not significant, Fig. 2A). Besides the four who now characterized their asthma as very mild, another considered its asthma as severe at recruitment. Additionally, 17 individuals perceived no change in their asthma severity. However, 28 individuals at recruitment and 30 individuals at follow-up had missing values for the asthma severity question. About asthma prescription medication, their use has increased in some categories between recruitment and follow-up. There was an increase of 31% in the number of individuals with asthma who take long-acting beta-agonists as their asthma medication combined in one inhaler with inhaled corticosteroids (ICSs) (χ^2^ p < 0.0001) but no significant changes were observed for those taking ICSs as a monotherapy or with an inhaler with short-acting beta-agonists or long-acting beta-agonists (Fig. 2B and Supplementary Table S2). New molecules have been developed over the past decades such as long-acting muscarinic antagonists and were added to the medication list of seven participants with asthma at follow-up. No targeted biologics were used by the Asthma group (anti-IgE, anti-interleukin (IL) 4 or anti-IL5).

**Figure 3:**
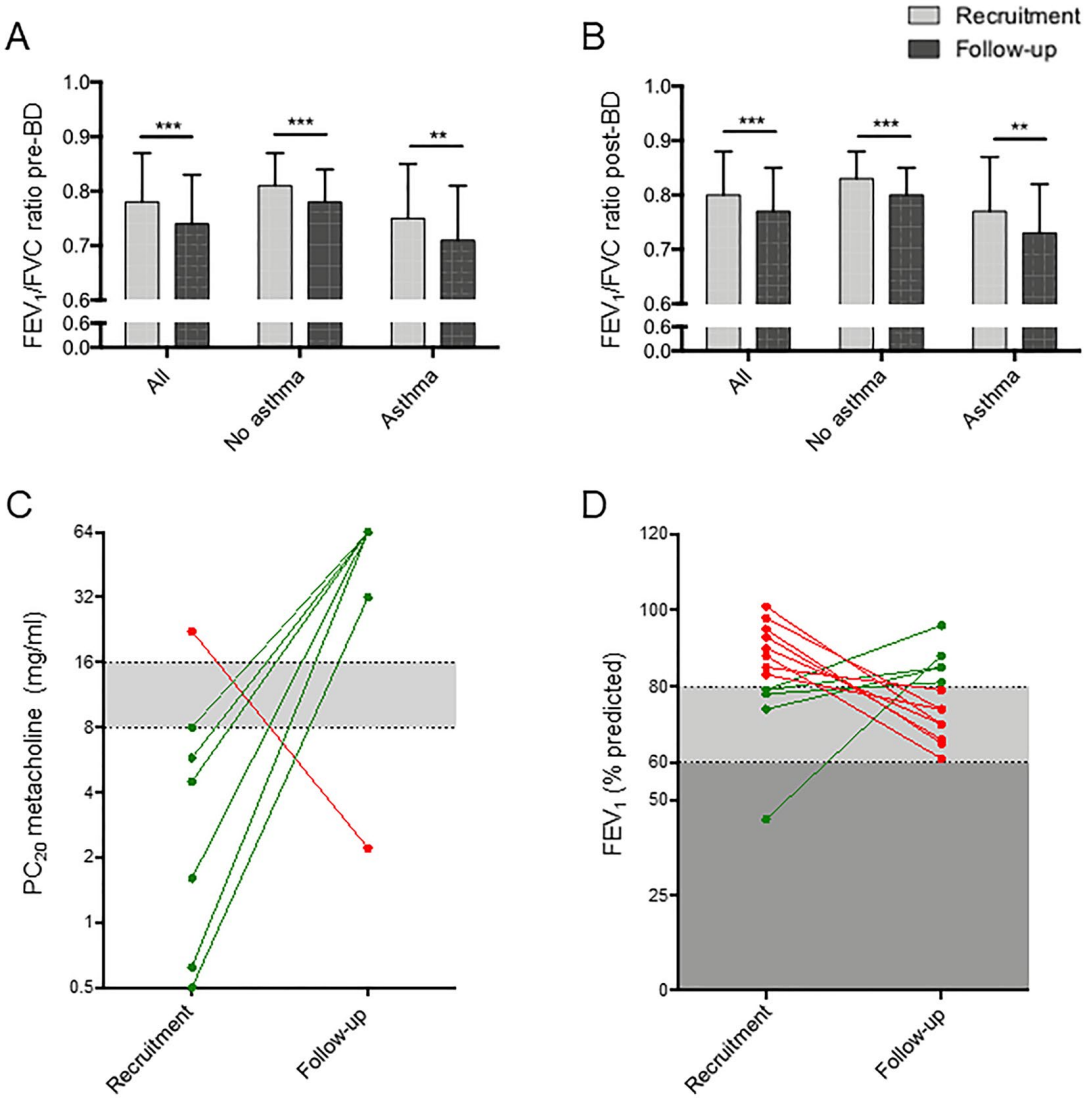
Variations in lung function. Changes in the forced expiratory volume in 1 s on the forced vital capacity (FEV_1_/FVC) ratio were observed between recruitment and follow-up considering values (**A**) before or (**B**) after the use of bronchodilators (BDs). The All group includes all participants in the subsample, the No asthma group comprises individuals without asthma at recruitment, and the Asthma group include participants with confirmed asthma diagnosis at recruitment. p values < 0.001 are indicated by two asterisks and p values < 0.0001 are indicated by three asterisks. (**C**) Representation of participants with asthma at recruitment who exhibited either an increase (from PC_20_ ≤ 8 mg/ml to ≥ 16 mg/ml; n = 7) or a decrease (from PC_20_ ≥ 16 mg/ml to ≤ 8 mg/ml; n = 1) in PC_20_ measurements. (**D**) Representations of participants with asthma at recruitment who showed an increase (from FEV_1_ < 80% to > 80%; n = 5) or a decrease (from FEV_1_ > 80% to < 80%; n = 8) in measurements of FEV_1_ of predicted values.

Significant decreases in the All group were observed for FEV_1_/FVC pre- and post-BD (3–4% of decline, p < 0.0001, Fig. 3A,B). Therefore, analyses were done to explain this decrease with other clinical parameters. Correlations between FEV_1_/FVC at follow-up and different lung parameters at recruitment and at follow-up were observed (Supplementary Table S3 and Supplementary Figs. S1–S3). Positive correlations were found with FEV_1_/FVC data at recruitment, FEV_1_ (% predicted) values at both recruitment and follow-up as well as log transformed PC_20_. It was also inversely correlated to BD response (% BD), log transformed IgE, percentage of circulating eosinophils at recruitment and follow-up, and percentage of circulating monocytes. Multiple linear regression models were also tested to explain its decrease with different sets of relevant clinical parameters. Those include baseline, disease diagnosis, respiratory, exposure and asthma treatment parameters for the independent variables at recruitment (see “Supplementary File” in the multiple linear regression section for complete list). The best significant multiple linear regression model was observed between FEV_1_/FVC pre-BD at follow-up and the percentage of BD change (% BD), the percentage of predicted value for FEV_1_ pre-BD (FEV_1_ pre-BD % pred) and the percentage of eosinophil count (% eosino) at recruitment (FEV_1_/FVC pre-BD at follow-up = 63.344 − 0.321 [% BD at recruitment] + 0.173 [FEV_1_ pre-BD % pred at recruitment] − 1.027 [% eosino at recruitment], R^2^ = 0.539, p = 2.82 × 10^−15^).

Results also showed a decrease in PC_20_ values in the No asthma group between the two visits (p** < **0.05, Table 1). Furthermore, an increase in PC_20_ values from < 8 to > 16 mg/ml of methacholine was noted for seven participants with asthma at recruitment and revealed the opposite for one individual (Fig. 3C). It was also observed that FEV_1_ values increased from < 80 to > 80% of predicted values for five participants with asthma and the opposite for eight others (Fig. 3D).

### Changes of COPD phenotype and other comorbidities

Based on COPD diagnoses validated by a physician and retrieved from the data collected at the archives of Chicoutimi Hospital, five participants developed COPD between recruitment and follow-up (Table 4). These were all individuals with asthma at recruitment and who were either smokers or ex-smokers and represent 7% of the Asthma group or 4% of all participants. At follow-up, two of these participants were obese, two overweight and one underweight, three had FEV_1_ percentage of predicted value < 80% and four had FEV_1_/FVC ratio < 0.70. Overall, 23 participants with asthma at follow-up had reduced FEV_1_/FVC ratio (< 0.70). In approximately half of these participants (n = 12), FEV_1_/FVC ratio was already below 0.70 at recruitment.

**Table 4:** Changes in the respiratory status of the five participants who developed a chronic obstructive pulmonary disease between recruitment and follow-up evaluation.

	Sex	Age of onset COPD	Age	BMI	Asthma	Atopy	Smoker	PC_20_	BD %	FEV_1_, %pred	FEV_1_/FVC
				Recruitment/follow-up							
1	M	66	58/74	31.96/35.99	Yes/yes	No/no	E-S/E-S	0.17/NA	0/36.36	27/33	0.57/0.68
2	M	63	54/70	25.06/29.06	Yes/yes	Yes/no	S/E-S	0.11/NA	NA/0	45/88	0.59/0.55
3	M	58	44/62	23.20/25.22	Yes/yes	Yes/yes	S/NA	0.06/1.90	19.00/10.30	70/68	NA/0.67
4	M	54	44/63	41.67/41.37	Yes/yes	Yes/yes	S/E-S	0.05/> 32	3.80/5.10	79/96	NA/0.75
5	F	52	51/69	17.04/15.64	Yes/yes	No/no	E-S/E-S	0.50/NA	18.87/16.67	53/36	0.63/0.56

*BMI* body mass index (kg/m^2^), *BD* bronchodilator response, *COPD* chronic obstructive pulmonary disease, *E-S* ex-smoker, *FEV*_*1*_ forced expiratory volume in 1 s, *FVC* forced vital capacity, *N-S* non-smoker, *NA* not available, *PC*_*20*_ provocative concentration for a 20% decrease in FEV_1_, *S* smoker.

BMI was significantly increased in all groups (p** < **0.0001 in All and Asthma group, p < 0.001 in No asthma group) at follow-up. Overall, individuals with asthma at follow-up were more likely to suffer from allergies (46% in the No asthma group compared to 57% in the Asthma group), to develop rhinitis comorbidity (17% compared to 30%) and to develop atopic dermatitis (40% compared to 48%; Table 1). Nevertheless, the incidence of atopy, rhinitis and atopic dermatitis decreased between recruitment and follow-up but was only significant for rhinitis (p** < **0.05; Table 1).

## Discussion

The objective of this study was to analyze the evolution of asthma and related phenotypes in time by assessing new diagnoses as well as changes in the respiratory condition and comorbidities of a subsample of the SLSJ asthma family cohort. When considering changes in asthma status, seven new cases of asthma in this cohort’s subsample were found, representing 12% of individuals without asthma at recruitment. This is in line with the literature. Indeed, adult onset of asthma occurs in approximately 10% of the population^17,18^. It is interesting to note that only one individual (#6) out of the seven has a profile in agreement with the Global Initiative for Asthma (GINA) guidelines for a non-asthmatic becoming asthmatic (atopy, methacholine PC_20_ < 8 mg/ml and > 12% reversibility in airway obstruction following bronchodilators)^4^.

### Atopy and BMI as risk factors of asthma

A study by Toskala et al. showed that atopy was a risk factor in the development and persistence of asthma^19^. This phenomenon was also observed in this study. Indeed, four of the seven individuals who developed asthma had allergies at recruitment and follow-up, and one developed atopy during this same period, representing 71% of the individuals with a new diagnosis of asthma. However, not all allergic asthma sufferers see their symptoms persist^19^. In fact, three individuals with asthma noticed their symptoms disappear and two of them were atopic at recruitment. The prevalence of allergic asthma phenotype considering all participants was persistent over time. The unchanged proportion of atopy phenotype in individuals with asthma at follow-up could be explained by the relatively stable concentration of IgE levels between the two periods (approximately 2.5 times higher than for individuals without asthma). This is consistent with the literature stating that deregulation of the immune system in allergic asthma results in elevated serum IgE levels^19^. It is remarkable that no significant change was observed for circulating IgE levels, regardless of age, asthma status, and medication, which evolved over the same period.

Another important risk factor is obesity^20^. Obesity is often associated with an increase in the severity of asthma, onset of asthma in adults, and difficult asthma control due to a decreased response to medication^5,21^. In the present study, an increase in BMI between recruitment and follow-up was observed in all groups. At follow-up, the average BMI was 27.06 kg/m^2^, which is considered overweight. However, the average BMI of individuals with a change in their respiratory status was greater than 30 kg/m^2^, which is considered obese. Four out of the seven individuals with adult-onset asthma had a BMI between 30 and 40 kg/m^2^. Therefore, these individuals presented a significant risk factor linked to their new respiratory status.

### Indices of asthma severity

The FEV_1_/FVC ratio is a respiratory parameter that determines the degree of airway obstruction^22^, with a value usually lower than < 0.75–0.80 for asthmatic individuals, and that decreases according to the increase in asthma severity^4^. In this study, the mean FEV_1_/FVC ratio decreased in both individuals with and without asthma over the years. Therefore, 54% of the decrease in FEV_1_/FVC pre-BD ratio at follow-up can be explained by a regression model in the SLSJ subsample that includes values at recruitment for the percentage of BD reversibility, the percentage of predicted value of FEV1 and the percentage of eosinophils. A comparable effect of blood eosinophil on the decline of lung function was found in a population-based cohort of young adults in New Zealand among participants aged from 21 to 38 years old^23^. The study determined that blood eosinophil counts were associated with a decrease of FEV_1_/FVC ratio including participants with and without asthma^23^. Additionally, in a population-based birth cohort that was followed into adulthood (until 26 years of age), a similar decrease in lung function in all groups, regardless of wheezing, sex, or other parameters were reported^24^. It would therefore be normal to observe a decrease in lung capacity given the two decades between the two measurements.

The FEV_1_/FVC values reflected the results of self-reported asthma severity in the questionnaire at follow-up. Individuals with asthma had an average FEV_1_/FVC which corresponds to mild asthma according to the literature^4^, and the majority classified their asthma as very mild or mild. The severity of asthma can also be determined by the class of medication taken to relieve symptoms and exacerbations^4^. The medication prescribed in the subsample is primarily short-acting beta-agonists and ICSs, which also correspond to mild asthma^4^. However, there has been a marked increase (31%) in the prescription of long-acting beta-agonists, usually prescribed to people with moderate asthma^4^, and the prescription of ICSs (11%) at follow-up. The changes in respiratory medication use could be due to the modifications in the GINA guidelines since recruitment; in 2019 ICS-formoterol was recommended in intermittent asthma and mild persistent asthma^4^. Whereas in the 2002 GINA report ICSs were recommended to be introduced in mild persistent asthma only^4^. This could indicate that the symptoms are well controlled by the medication, leading to a self-reported asthma severity by the participants as very mild and mild.

Smoking has a significant impact on asthma, for example through epigenetic changes^3^. The number of smokers significantly decreased (from 22 to 4) between recruitment and follow-up. However, the risk of developing asthma in adulthood due to this exposure remains^3^. For individuals who had a new status of asthma at follow-up, five out of seven participants were either ex-smokers or smokers. Moreover, 58% of the 24 individuals with adult-onset asthma at recruitment were either ex-smokers or smokers. Among the 24 individuals at recruitment and the 7 individuals at follow-up with adult-onset asthma, 4 have developed obstructive sleep apnea and 3 have or had psychological disturbances (depression and anxiety disorder). These comorbidities can be found in people with severe asthma as can gastro-esophageal reflux disease and chronic infections and may be associated with difficult asthma outcomes^25,26^. Respiratory infections noted for these participants were not recurrent or chronic. Since the SLSJ asthma family cohort is mainly comprised of participants with mild-to-moderate asthma it is expected to have little comorbidities in the subsample.

### Development of COPD among asthmatic participants

A study by Mirabelli et al. demonstrated the presence of COPD in 29% of a population with a history of asthma^27^. Individuals with asthma who smoked are more likely to develop COPD as they age and thus may develop asthma-COPD overlap (ACO) phenotypes^3,28^. In this study, five participants who were asthmatic at recruitment (7%) developed COPD and all of them were ex-smokers or smokers. For all of them, PC_20_ was also very low at recruitment, four of the five individuals who developed COPD had values less than 0.25 mg/ml which corresponds to severe AHR, and one had a value of 0.5 mg/ml, which is considered moderate AHR^29^. This suggests that this risk factor may have contributed to the development of COPD^30,31^. These results contribute to a better understanding of ACO which is critical since patients affected by both diseases have more exacerbations, poorer quality of life, and faster decline in lung function, as well as a higher death rate than patients with only asthma or COPD^22,32–35^.

## Strength and limitations

The major strength of this study is the fine phenotyping performed both at recruitment and follow-up for respiratory diseases’ diagnoses as well as respiratory measures, comorbidities, and risk factors. A possible limitation of this study is its complexity. There are a large number of symptoms, environmental exposures, lifestyle habits, and comorbidities related to the heterogeneity of this disease^36^. In addition, the conception of a longitudinal study lasting 20 years after the first recruitment bears some difficulties. Thus, another limitation is due to change over time of knowledge about causes and treatments of diseases, leading to new parameters that are not comparable between the two-time points. For instance, in 1997 the standardized questionnaire used in this study at recruitment did not include questions assessing the non-pharmacological interventions to control asthma symptoms. Nor did it have questions helping determine the participants medical care satisfaction or adherence to their asthma treatments, making it impossible to incorporate those parameters in the longitudinal study. Furthermore, a memory bias from the participants may be introduced in some data from the questionnaire, as to assess when the changes in the asthma status occurred, since there is a long period between recruitment and follow-up. In order to prevent from this same kind of memory bias on other phenotypic parameters, clinical parameters that can be measured or diagnosed were prioritized in analyses. No bias about asthma sub-phenotypes was introduced in this study. In fact, a total of 215 participants were selected in the context of another project and the follow-ups were done at the same time. The only criteria for the selection of participants in the other project was to have one asthmatic and one non-asthmatic individual per family. The selection of the participants did not take into account the severity nor the type of asthma. A larger subsample of the cohort may provide a better representation of asthma over time. Moreover, difficulties linked to the recontact of participants make longitudinal studies more difficult to design as also found in other longitudinal studies^11,36^.

## Conclusion

The 20-year follow-up of a subsample from the SLSJ asthma family cohort presented new asthma status for 12% of the participants without asthma at recruitment as well as COPD development for five individuals, with a description of risk factors for these individuals. Longitudinal studies like this one will allow a better characterization of asthma and comorbidities over time. One perspective of this type of study is to assess phenotypic changes and link them to the genetic and epigenetic signatures in order to understand the genetic mechanisms involved.

## Supplementary Information

Supplementary Information.

## Data Availability

Datasets used or analyzed during the current study are available from the corresponding author on a reasonable request.

## Abbreviations

ACO: Asthma-COPD overlap
AHR: Airway hyperresponsiveness
BD: Bronchodilator
BMI: Body mass index
CIUSSS: Centre Intégré Universitaire de Santé et de Services Sociaux
COPD: Chronic obstructive pulmonary disease
FEV_1_: Forced expiratory volume in 1 s
FVC: Forced vital capacity
ICS: Inhaled corticosteroids
IgE: Immunoglobulin E
IL: Interleukin
PC_20_: Methacholine concentration inducing a 20% decrease in FEV_1_
SLSJ: Saguenay–Lac-Saint-Jean
T_H_2: T-helper type 2 cells
