# Supplementary material for: Longitudinal follow-up of the asthma status in a French–Canadian cohort
Source: Sci Rep. 2022 Aug 13;12:13789. doi: 10.1038/s41598-022-17959-6 (PMC9376060; doi:10.1038/s41598-022-17959-6)
Supplement: Supplementary file 1 — Supplementary Information. [file 41598_2022_17959_MOESM1_ESM.docx]

**Longitudinal follow-up of the asthma status in a French-Canadian cohort**

Marie-Eve Lavoie, Jolyane Meloche, Anne-Marie Boucher-Lafleur, Paul Bégin, Charles Morin, Louis-Philippe Boulet, Anne-Marie Madore, Catherine Laprise.

**Supplementary Information File**

**MATERIALS AND METHODS**

**Study population**

Recruitment was conducted through probands who had at least two of the following criteria: (1) at least three clinic visits for acute asthma within 1 year; (2) two or more asthma-related hospital admissions within 1 year; or (3) use of steroids to control symptoms (6 months of oral or 1 year of inhaled corticosteroids [ICS] use). We then included all family members who wanted to participate in the study, with both parents (father and mother) being included. Furthermore, one of the parents had to be unaffected, and all four grandparents had to be of French-Canadian origin. A fine phenotypic profile, including more than 75 phenotypic traits, was defined for each participant using a standardized questionnaire, immunological measure, spirometry, bronchial (methacholine) challenge, allergic tests, and blood cell counts. A complete description of the cohort at recruitment can be found in Laprise [14].

**Data collection**

Allergy was assessed using skin prick test for 27 allergens including common aeroallergens from animals (cat, dog, cow and horse dander, and bird feathers), indoor aeroallergens (*Dermatophagoides farinae* and *D. pterionisus*), and outdoor aeroallergens (ryegrass, weeds, ambrosia, timothy hay, grasses, tree mix, maple, birch, oak, elm, *Cladosporium*/*Hormodendrum*, *Alternaria*, *Aspergillus,* and *Penicillium*), as well as food allergens (rye, oat, barley flours, peanuts, whole wheat, and egg whites).

**Multiple linear regression**

List of relevant clinical parameters tested in the multiple linear regression models as independent variables: baseline parameters at recruitment (sex, age, BMI), disease diagnosis parameters at recruitment (asthma, age of onset of asthma, asthma severity, atopy, rhinitis, atopic dermatitis), respiratory parameters at recruitment (FEV_1_ pre-BD, FEV_1_/FVC pre- and post-BD, PC_20_, BD reversibility), white blood cell counts at recruitment (percentage of eosinophils, lymphocytes, monocytes, neutrophils, basophils), smoking status at recruitment and asthma treatment at recruitment (short-acting β2-agonist, long-acting β2-agonist, inhaled corticosteroids, oral corticosteroids, leukotriene modifiers, methylxanthines).

**Supplementary Tables**

**Table S1.** Self-reported asthma severity of 125 individuals at recruitment and follow-up

|  | **Recruitment** | | | **Follow-up** | | |
| --- | --- | --- | --- | --- | --- | --- |
| **Parameter** | All participants  (n=125) | No asthma  (n=57) | Asthma  (n=68) | All participants*  (n=125) | No asthma*  (n=57) | Asthma*  (n=68) |
| **Very mild, n (%)** | 2 (2)^†^ | 0 | 2 (3)^†^ | 14 (11)^†^ | 2 (4) | 12 (18)^†^ |
| **Mild, n (%)** | 17 (14) | 0 | 17 (25) | 18 (14) | 2 (4) | 16 (24) |
| **Moderate, n (%)** | 16 (13) | 0 | 16 (24) | 11 (9) | 1 (2) | 10 (15) |
| **Severe, n (%)** | 4 (3) | 0 | 4 (6) | 2 (2) | 0 | 2 (3) |
| **Very Severe, n (%)** | 1 (1) | 0 | 1 (2) | 0 | 0 | 0 |
| **Missing values, n** | 28 | 0 | 28 | 30 | 2 | 28 |

*Same individuals as at recruitment

Statistics were performed between recruitment and follow-up, Chi-square test or Fisher exact test for All, No asthma and Asthma groups: †p **<** 0.05.

**Table S2.** Asthma medication use of 125 individuals at recruitment and follow-up

|  | **Recruitment** | | | **Follow-up** | | |
| --- | --- | --- | --- | --- | --- | --- |
| **Parameter** | All participants  (n=125) | No asthma  (n=57) | Asthma  (n=68) | All participants*  (n=125) | No asthma*  (n=57) | Asthma*  (n=68) |
| **Short-acting β2-agonist, n (%)** | 50 (40) | 0^†^ | 50 (74) | 52 (42) | 9 (16)^†^ | 43 (63) |
| **Long-acting β2-agonist, n (%)** | 6 (5)^†††^ | 0 | 6 (9)^†††^ | 31 (25)^†††^ | 4 (7) | 27 (40)^†††^ |
| **Inhaled corticosteroids, n (%)** | 41 (33)^†^ | 0^††^ | 41 (60) | 58 (46)^†^ | 10 (18)^††^ | 48 (71) |
| **Oral corticosteroids, n (%)** | 2 (2) | 0 | 2 (3) | 3 (2) | 0 | 3 (4) |
| **Leukotriene modifiers, n (%)** | 2 (2)^†^ | 0 | 2 (3)^†^ | 11 (9)^†^ | 2 (4) | 9 (13)^†^ |
| **Methylxanthines, n (%)** | 3 (2) | 0 | 3 (4) | 1 (1) | 0 | 1 (2) |
| **Long-acting muscarinic antagonists, n (%)** | 0^†^ | 0 | 0^†^ | 7 (6)^†^ | 0 | 7 (10)^†^ |

*Same individuals as at recruitment

Statistics were performed between recruitment and follow-up, Chi-square test or Fisher exact test for All, No asthma and Asthma groups: †p **<** 0.05, †† p < 0.001 and †††p < 0.0001.

**Table S3.** Significant correlations with FEV_1_/FVC ratio at follow-up

| **Variable** | ***Statistics of correlations with variables at recruitment** | | ***Statistics of correlations with**  **variables at follow-up** | |
| --- | --- | --- | --- | --- |
|  | r | p | r | p |
| **FEV_1_, % pred** | 0.508 | 1.729×10^-8^ | 0.548 | 7.126×10^-10^ |
| **FEV_1_/FVC pre-BD** | 0.731 | 8.645×10^-19^ | NA | NA |
| **FEV_1_/FVC post-BD** | 0.633 | 3.059×10^-10^ | 0.914 | 7.645×10^-43^ |
| **%BD** | -0.497 | 1.629×10^-7^ | -0.497 | 3.738×10^-8^ |
| **Log PC_20_** | 0.397 | 2.815×10^-5^ | 0.375 | 3.141×10^-4^ |
| **Log IgE** | -0.249 | 1.011×10^-2^ | -0.292 | 2.387×10^-3^ |
| **Eosinophils (%)** | -0.365 | 1.491×10^-4^ | -0.347 | 5.243×10^-4^ |
| **Monocytes (%)** | -0.219 | 2.530×10^-2^ | -0.213 | 3.610×10^-2^ |

*Spearman Rho correlations were performed between FEV_1_/FVC at follow-up and variables at recruitment.

Definition of abbreviations: BD = bronchodilator response; FEV_1_ = forced expiratory volume in 1 sec; FVC = forced vital capacity; IgE = immunoglobulin E; NA = not available; PC_20_ = provocative concentration for a 20% decrease in FEV_1_.

**Supplementary Figures**


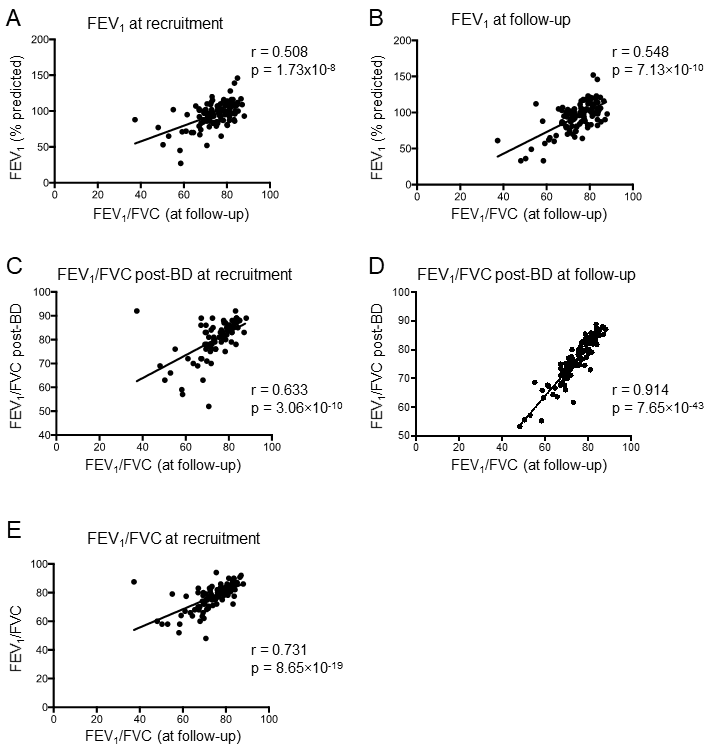


**Figure S1. Associations between FEV_1_/FVC at follow-up and other lung function parameters**

Spearman Rho correlations were performed between FEV_1_/FVC at follow-up (pre-BD), and FEV_1_ expressed in % of predicted values at (A) recruitment and (B) follow-up. FEV_1_/FVC at follow-up strongly correlated with FEV_1_/FVC post BD use at both (C) recruitment and (D) follow-up and a strong correlation was observed between (E) FEV_1_/FVC ratio at recruitment and follow-up.

BD = bronchodilator; FEV_1_ = forced expiratory volume in 1 sec; FVC = forced vital capacity.


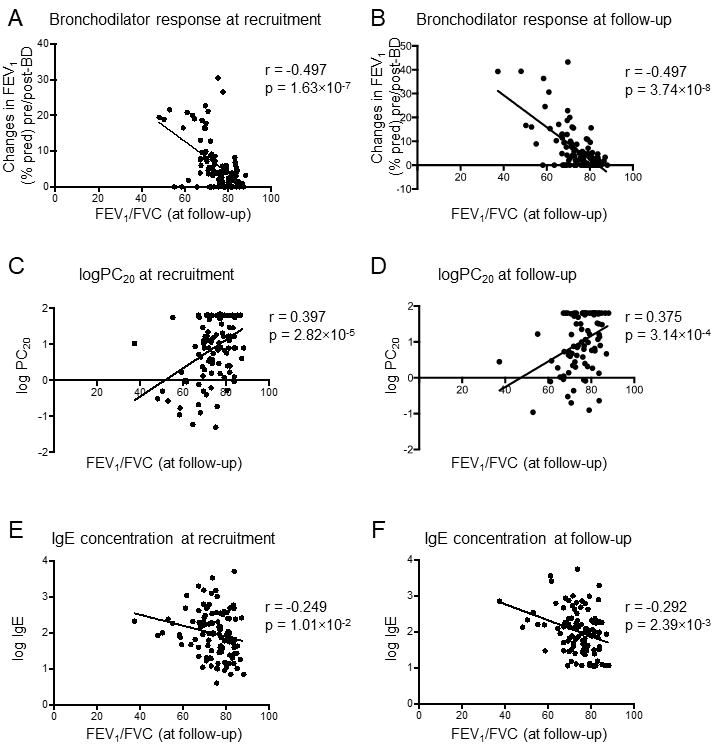


**Figure S2. Associations between FEV_1_/FVC at follow-up and other respiratory or clinical measurements**

Spearman Rho correlations were performed between FEV_1_/FVC at follow-up and BD response at both recruitment (A) and follow-up (B). The FEV_1_/FVC ratio was also correlated to values of log transformed PC_20_ (methacholine challenge) at both recruitment (C) and follow-up (D). Finally, FEV_1_/FVC at follow-up was also significantly associated with log transformed circulating IgE levels at both recruitment (E) and follow-up (F), although these correlations were weak.

BD = bronchodilator; FEV_1_ = forced expiratory volume in 1 sec; FVC = forced vital capacity; IgE = immunoglobulin E; PC_20_ = provocative concentration for a 20% decrease in FEV_1_.


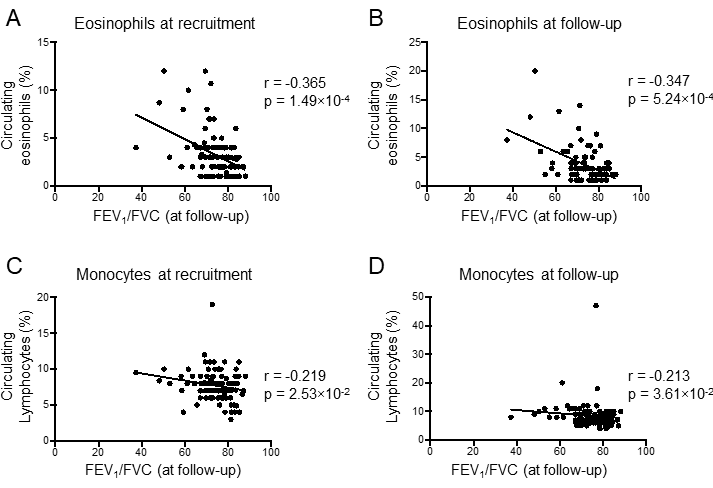


**Figure S3. Associations between FEV_1_/FVC at follow-up and circulating inflammatory cells**

Spearman Rho correlations were performed between FEV_1_/FVC ratios at follow-up and circulating levels (%) of eosinophils at recruitment (A) and follow-up (B), as well as circulating levels (%) of monocytes at both recruitment (C) and follow-up (D).

FEV_1_ = forced expiratory volume in 1 sec; FVC = forced vital capacity.
